# Prostate cancer and the unfolded protein response

**DOI:** 10.18632/oncotarget.9912

**Published:** 2016-06-09

**Authors:** Margrethe Storm, Xia Sheng, Yke Jildouw Arnoldussen, Fahri Saatcioglu

**Affiliations:** ^1^ Department of Biosciences, University of Oslo, Oslo, Norway; ^2^ Institute for Cancer Genetics and Informatics, Oslo University Hospital, Oslo, Norway; ^3^ Department of Biological and Chemical Work Environment, National Institute of Occupational Health, Oslo, Norway

**Keywords:** endoplasmic reticulum stress, prostate cancer, unfolded protein response, therapeutic targeting

## Abstract

The endoplasmic reticulum (ER) is an essential organelle that contributes to several key cellular functions, including lipogenesis, gluconeogenesis, calcium storage, and organelle biogenesis. The ER also serves as the major site for protein folding and trafficking, especially in specialized secretory cells. Accumulation of misfolded proteins and failure of ER adaptive capacity activates the unfolded protein response (UPR) which has been implicated in several chronic diseases, including cancer. A number of recent studies have implicated UPR in prostate cancer (PCa) and greatly expanded our understanding of this key stress signaling pathway and its regulation in PCa. Here we summarize these developments and discuss their potential therapeutic implications.

## THE ENDOPLASMIC RETICULUM (ER)

The ER is an eukaryotic organelle arranged in a tubular network which is involved in the synthesis, folding, and trafficking of proteins, as well as being a key site for intracellular Ca^2+^ homeostasis. The highly oxidative, calcium-rich ER lumen facilitates the genesis of proteins destined for secretion or targeted to transmembrane compartments, constituting approximately 30% of the total proteome in eukaryotic cells. In addition, the ER regulates a variety of metabolic processes, such as gluconeogenesis and lipid biosynthesis, as well as biogenesis of autophagosomes and peroxisomes [1].

The ER is not only vital for these key cellular functions, but it also serves as a homeostatic device that monitors the intracellular environment and adjusts metabolic and stress responses accordingly. Stressful conditions such as an accumulation of unfolded proteins (e.g. when the demand for protein secretion is high), imbalance in ER Ca^2+^ levels, glucose deprivation, or hypoxia can all lead to disruption of ER function termed ER stress [2]. In an attempt to restore ER homeostasis and normal cellular function, several signal transduction pathways are activated. However, if the stress cannot be resolved, the pro-survival signaling switches to a pro-apoptotic one resulting in cell death. Both of these possible outcomes are mediated by the regulation of highly integrated signal transduction pathways, collectively called the unfolded protein response (UPR) [1].

## THE UNFOLDED PROTEIN RESPONSE

**Figure 1:**
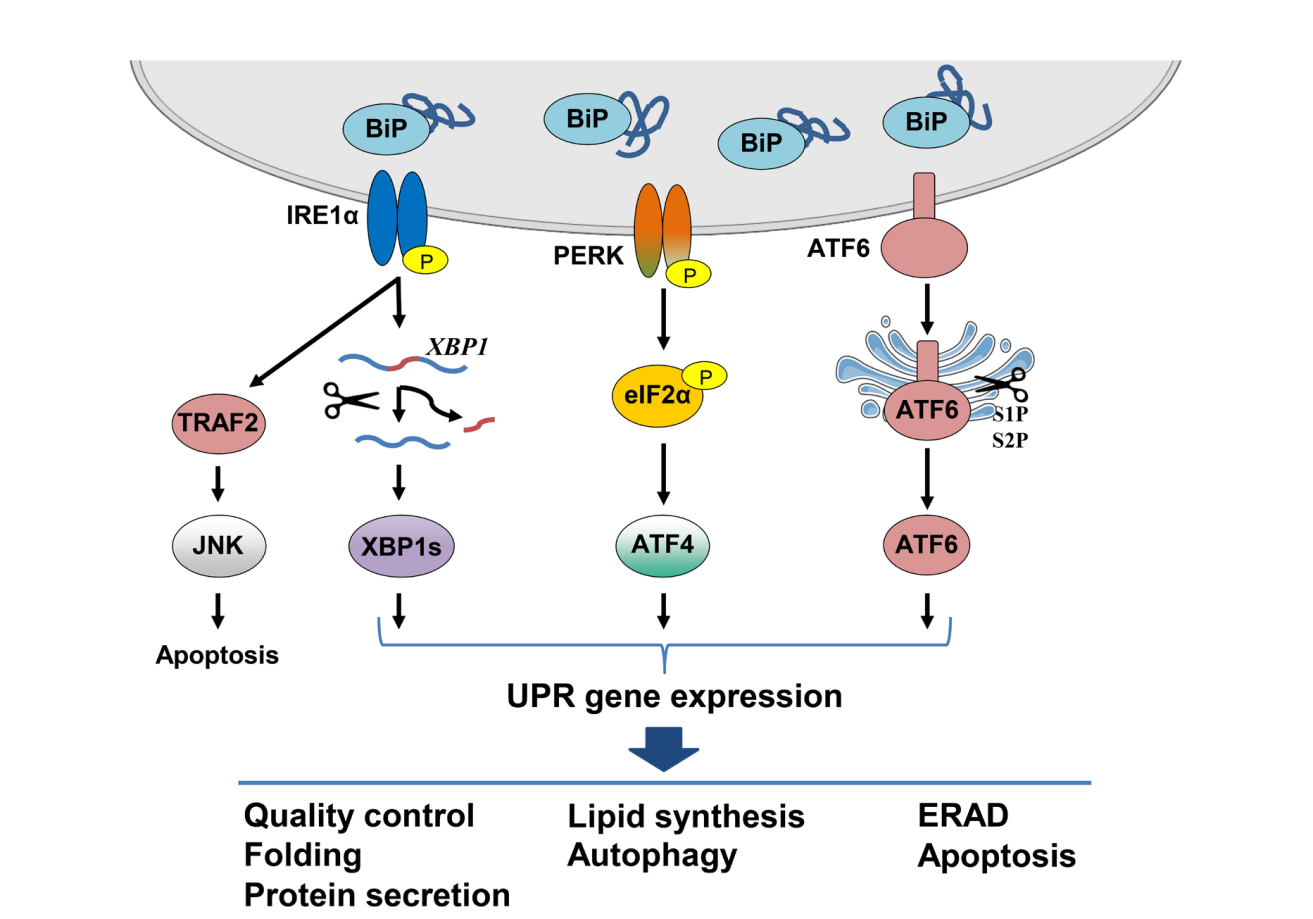
Schematic representation of the canonical UPR signaling pathways The UPR is activated by the accumulation of unfolded proteins in the ER lumen as BiP dissociates from the three ER stress sensors IRE1α, ATF6α and PERK. Oligomerization of IRE1α leads to its activation and the generation of the transcription factor XBP1s which translocates to the nucleus and induces expression of genes whose products are involved in protein folding and ERAD. Additionally, IRE1α activation leads to degradation of ER-associated mRNAs through RIDD and induces JNK signaling, both of which result in induction of apoptosis. Activated ATF6α is cleaved in the Golgi by the S1P and S2P proteases to produce a transcription factor which translocates to the nucleus and induces chaperone gene expression. UPR signaling leads to a translational block through the PERK mediated phosphorylation of eIF2α. Despite the inhibition of global protein synthesis, ATF4 is translated, leading to the induction of genes involved in autophagy and amino acid metabolism. Signaling through the UPR aims to restore ER homeostasis by blocking further build-up of unfolded proteins, enhancing the folding capacity and initiating degradation of misfolded proteins. Upon persistent ER stress, however, pro-apoptotic signaling is induced and the cell undergoes programmed cell death.

In multicellular eukaryotes the canonical UPR pathways are initiated by three proteins that reside in the ER membrane: inositol requiring-enzyme 1 alpha (IRE1α), activating transcription factor 6 alpha (ATF6α) and protein kinase RNA-like ER kinase (PERK). Upon activation, these ER ‘sensors’ initiate signaling cascades that elicit corrective actions to restore ER homeostasis [1] (Figure 1). Under normal physiological conditions, these transmembrane proteins are held in an inactive configuration by BiP. Upon accumulation of misfolded proteins, or other stimuli that can activate the UPR, BiP dissociates from the ER sensors and binds instead to unfolded proteins in the ER lumen. This results in IRE1α and PERK oligomerization leading to their phosphorylation, as well as translocation of ATF6α to the Golgi where it is cleaved and becomes an active transcription factor. Activation of IRE1α and PERK signaling also activates downstream transcription factors (see below) leading to changes in gene expression and corresponding phenotypic responses in the cell.

UPR signaling aims to restore homeostasis by inducing expression of proteins involved in almost every aspect of the secretory pathway. Gene expression data has demonstrated that the UPR is involved in protein entry into the ER, folding, glycosylation, ER-associated degradation (ERAD), protein quality control, redox metabolism, autophagy, lipid biogenesis and vesicular trafficking [1, 2].

## IRE1

IRE1 is both a site-specific endoribonuclease and a Ser/Thr kinase; in mammals, it exists as two isoforms, IRE1α and IRE1β. IRE1α is expressed in all tissues, whereas IRE1β is only found primarily in the gastrointestinal and respiratory tracts, and thus the α isoform has been more extensively studied [3]. In response to unfolded proteins in the ER lumen, IRE1α dimerizes and oligomerizes, resulting in trans-autophosphorylation of the kinase domains. This leads to activation of the cytosolic RNase domain and the highly sequence specific endoribonucleolytic cleavage and subsequent splicing of the mRNA encoding a transcription factor called X-box binding protein 1 (XBP1) [4]. IRE1α excises a 26-nucleotide-long intron in the *XBP1* mRNA that results in a frame shift giving rise to an active and stable transcription factor termed spliced XBP1 (XBP1s). When translocated to the nucleus, XBP1s induces UPR target gene expression [4]. The mammalian ligase responsible for joining the two exons of *XBP1* mRNA upon removal of the intron, RtcB, was recently identified [5]. In contrast to the spliced form, the unspliced XBP1 (*XBP1u*) encodes a protein that is more labile, represses UPR target genes, and negatively regulates XBP1s and ATF6α by promoting their degradation [6].

XBP1s alone, or in conjunction with other transcription factors, launches a transcriptional program that activates the production of chaperones, proteins involved in ER biogenesis, phospholipid synthesis that is required for ER expansion under ER stress, ERAD, and secretion (e.g. ER degradation-enhancing alpha-mannosidase-like 1 (EDEM), ER-localized DnaJ 4 (Erdj4), and protein disulfide isomerase (PDI)) [7]. IRE1α-XBP1s signaling is therefore one of the major pathways for enhancing the folding capacity of the ER and coping with ER stress.

*XBP1* mRNA was first thought to be the only known substrate for IRE1α; however, IRE1α was later found to also control gene expression *via* an XBP1-independent post-transcriptional mechanism [8]. Notably, the IRE1α ribonuclease activity can, under certain conditions, target and degrade other ER-associated mRNAs in a process termed regulated IRE1-dependent decay (RIDD) [8-10]. A number of mRNAs as well as miRNAs involved in different cellular processes have been shown to be targeted by the RIDD activity of IRE1α resulting in different cellular outcomes [11, 12]. It has been suggested that the RIDD activity of IRE1α was dependent on the oligomeric state of IRE1α [13]. In contrast, a recent study in yeast proposed the opposite view where RIDD is favored when yeast IRE1 acts as a monomer/dimer, while oligomerization promotes splicing of the mRNA for yeast homologue of XBP1, HAC1 [14].

Phosphorylation and activation of IRE1α also leads to the recruitment of the adaptor protein tumor necrosis factor receptor (TNFR)-associated factor 2 (TRAF2) and apoptosis signal regulating kinase 1 (ASK1) to the cytoplasmic leaflet of the ER membrane. This initiates a cascade of phosphorylation events that result in the activation of c-Jun N-terminal kinase (JNK) [15, 16]. As JNK activity has been closely linked to cell death [17-19], this connects ER stress-induced IRE1α signaling to apoptosis under certain settings.

## ATF6

ATF6α and ATF6β are ER stress transducers that belong to the basic leucine zipper (bZIP) family of transcription factors [1]. In response to stress, ATF6 translocates to the Golgi, where it is processed by site-1 proteases (S1P) in its ER luminal domain and by site-2 proteases (S2P) within the region spanning the Golgi bilayer (Figure 1). This releases a cytosolic fragment which then translocates to the nucleus and functions as a transcription factor that binds to ER stress response elements (ERSE) in target genes [20]. The dissociation of BiP from ATF6α in response to stress within the ER lumen is proposed to unmask a Golgi-localization signal in the protein, allowing it to react with COPII, and be transported to the Golgi [21]. There is also evidence for calreticulin involvement in ATF6 transport from the ER to the Golgi, since under-glycosylated ATF6 as a result of ER stress did not interact with calreticulin which led to its transport to the Golgi for processing [22]. Furthermore, a recent study has suggested that PERK signaling is involved in regulating ATF6α trafficking and thus its activation, underlying the crosstalk between the canonical UPR arms [23]. In addition, the protein disulfide isomerase A5 (PDIA5) has been linked to ATF6 activation upon ER stress [24].

Target genes of ATF6α include chaperones BiP and GRP94, ERAD components and the UPR genes XBP1, protein kinase inhibitor of 58 kDa (P58IPK/DNAJC3), and C/EBP homologous protein (CHOP/GADD153) [4, 25-28]. ATF6α can also heterodimerize with XBP1s to regulate transcription from UPR elements (UPRE) in target genes, another example of crosstalk between UPR arms [26]. Similar to ATF6α, ATF6β is cleaved and translocates to the nucleus upon ER stress. However, ATF6β is a poor transcriptional activator and appears to repress ATF6α-mediated induction of UPR targets [29].

## PERK

The third canonical UPR sensor is PERK, an ER transmembrane Ser/Thr kinase that attenuates translation in response to ER stress [30]. Upon accumulation of unfolded proteins in the ER lumen, PERK dimerizes, autophosphorylates and subsequently phosphorylates Ser51 in eukaryotic translation initiation factor 2 (eIF2) α-subunit resulting in attenuation of global translation [31] (Figure 1). The decrease in global translation quickly reduces the amount of newly synthesized proteins entering the ER, enabling it to recover. Despite a halt in translation, a few selected mRNAs with short upstream open reading frames (uORF) in the 5′-UTR escape this translational inhibition [31]. The best characterized example of this in mammals is activating transcription factor 4 (ATF4), which regulates the expression of genes involved in redox balance, amino acid metabolism, protein folding, autophagy and cell survival [2, 32]. Among the ATF4 target genes is *CHOP,* encoding a transcription factor involved in regulation of apoptosis [1]. CHOP has been shown to promote apoptosis demonstrated by *Chop*-/- MEFs displaying enhanced cell survival when treated with ER stressors compared to wild type cells [33]. CHOP can trigger apoptosis possibly through the transcriptional induction of proapoptotic BIM and the downregulation of antiapoptotic BCL-2 expression leading to apoptosis [34, 35]. PERK signaling is fine-tuned by the CHOP target gene growth arrest and DNA damage-inducible 34 (GADD34) which associates with the phosphatase PP1 and promotes dephosphorylation of eIF2α, thereby alleviating translational inhibition [1, 2]. Thus, PERK signaling is central in the switch between the adaptive response phase and chronic ER stress leading to apoptosis. In addition, CHOP promotes oxidative protein folding in the ER through the induction of ER oxidoreductin-1 alpha (ERO1α) expression. However, the resulting increase in disulphide bond formation generates reactive oxygen species (ROS) [36, 37]. This might suggest that under conditions of chronic ER stress the CHOP-mediated increase in protein flux into the ER through GADD34, and the subsequent increase in ROS formation can in turn lead to an enhanced stress leading to apoptosis [37, 38].

UPR signaling can counter the effect of ROS by launching an anti-oxidant response. PERK activates the transcription factors ATF4 and nuclear factor E2 related factor 2 (NRF2), which induce genes involved in anti-oxidation [1]. NRF2 is held in the cytoplasm through its association with KEAP; phosphorylation of NRF2 by PERK triggers its dissociation and the nuclear import of NRF2 [39]. PERK-eIF2α signaling also activates NF-κB through translational repression of inhibitor of kappa B (IκB), resulting in regulation of apoptosis [40, 41]. The co-chaperone P58IPK, a target of both XBP1s and ATF6α, inhibits PERK signaling by interacting with the kinase domain of PERK and impairing eIF2α phosphorylation [42-44]. This is another example of the self-controlling, auto-corrective nature of the UPR response.

Another aspect adding to the complexity is that UPR signaling through the three canonical branches induce the expression of various miRNAs. Conversely, a number of miRNAs have been shown to target UPR components, [45, 46]. This layer of regulation is another reminder that the UPR needs to be regulated in multiple ways in a manner that allows versatile response to ques from the environment.

Another example of this is that in humans, there are three other eIF2α kinases that can phosphorylate eIF2α and regulate translation: General control non-derepressible-2 (GCN2) which is activated by nutrient deprivation, heme-regulated initiation factor 2 alpha kinase (HRI) activated by heme deficiency and oxidative stress, and protein kinase interferon-inducible double stranded RNA dependent (PKR) which is activated by viral infection. They all converge on the phosphorylation of the same residue in eIF2α and are collectively referred to as the integrated stress response (ISR) [1, 36].

**Figure 2:**
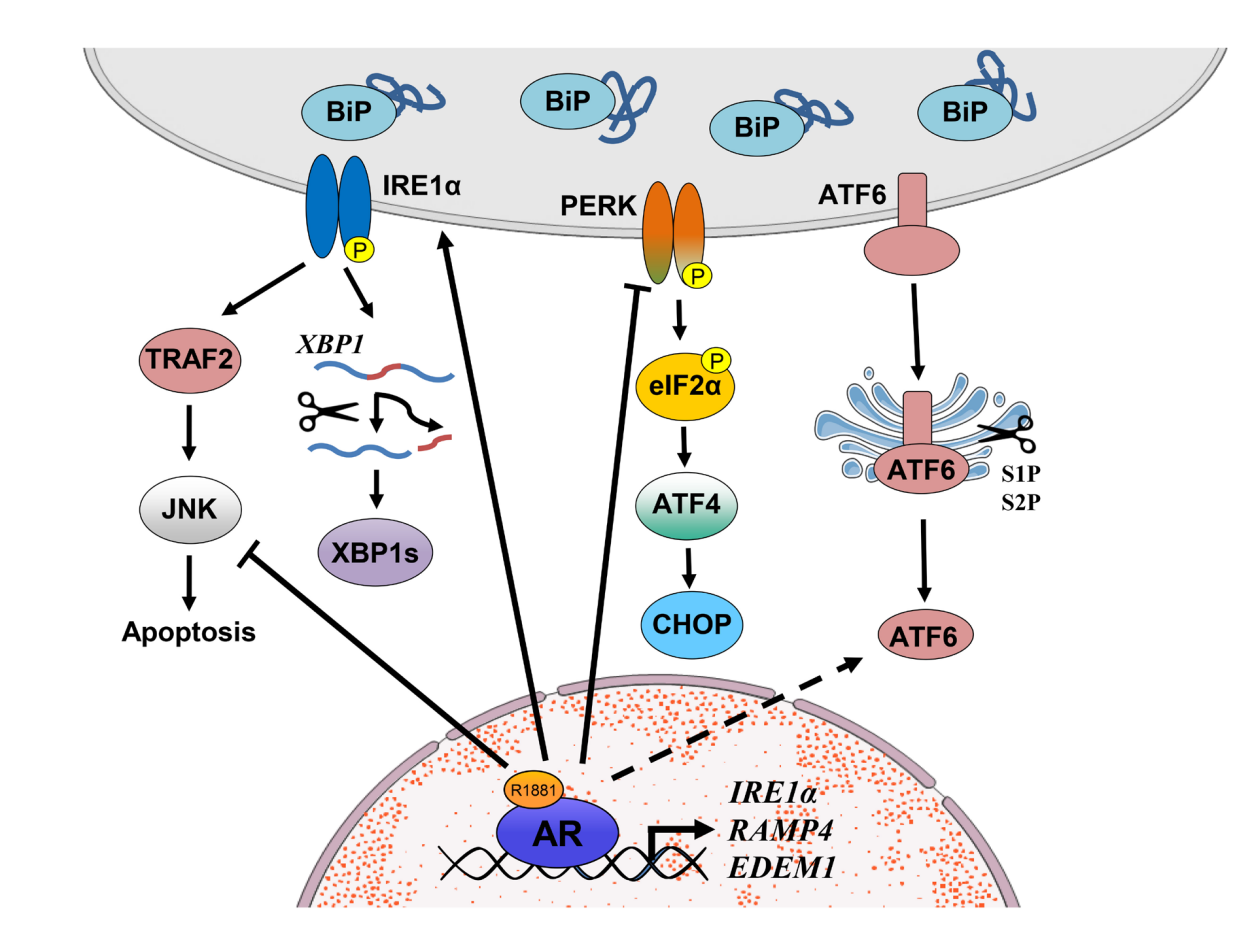
Schematic representation of UPR signaling in prostate cancer Liganded AR binds in the vicinity of the IRE1α, RAMP4 and EDEM1 genes to increase their expression, thus activating the IRE1α pathway but at the same time inhibits pro-apoptotic JNK signaling which is also activated by IRE1α. In contrast, androgens inhibit PERK and eIF2α activity. However, contrary to expectations (see text), ATF4 and CHOP expression are modestly increased in response to androgen; thus, the exact role of the PERK pathway and its regulation by androgens in prostate cancer requires further investigation. It is currently unknown whether the ATF6 pathway is regulated by androgen signaling in prostate cancer cells. Arrows with solid lines indicate a direct established effect whereas those with dashed lines indicate indirect or currently unexplored interactions.

## UPR IN CANCER

Solid tumors and their metastatic derivatives, often experience hypoxia, nutrient deprivation, lactic acidosis, and oxidative stress which compromise ER function leading to ER stress. In many respects the UPR can be viewed as a cytoprotective mechanism, favoring tumor growth by enabling cancer cells to survive under very unfavorable conditions. However, from another perspective, UPR can serve to protect the host by initiating cell death pathways [2]. UPR signaling has been implicated in various aspects of the tumor cell biology including including angiogenesis, invasion, mitochondrial function, intercellular communication, and tumor-associated inflammation [2]. The connections between ER stress and UPR in oncogenesis and cancer development have been extensively reviewed elsewhere [2, 12, 32].

The majority of the studies in cancer cells so far point to UPR activation as an adaptive survival mechanism. For example, consistent with this notion, increased levels of GRP94 and BiP have been correlated with gastric cancer [47]. The IRE1α arm is also implicated in tumor tolerance to hypoxia and pro-angiogenetic mechanisms, as XBP1s was increased in hypoxic cells and provided a survival advantage [48]. However, despite the large amount of data implicating ER stress and UPR activation in many forms of cancer, it is still not clear whether UPR ultimately promotes or inhibits tumor growth.

UPR activation as a cytoprotective response is supported by the fact that XBP1 overexpression in cancer cells directly promotes tumorigenesis, such as in chronic lymphocytic leukemia [49]. In multiple myeloma (MM), targeting the activated IRE1α and subsequent *XBP1* splicing by a number of compounds has been tested with promising results in preclinical models. Furthermore, there were synergistic effects in combination with bortezomib, the FDA approved proteasome inhibitor for MM [50]. Another study demonstrated that XBP1s loss may confer MM cells resistance to bortezomib [51]. XBP1 interacts with hypoxia-inducible factor 1-alpha (HIF1α) in triple negative breast cancer and drives tumor progression by inducing hypoxia signature gene expression [52]. Interestingly, a recent study also showed that XBP1 can blunt the antitumor activity by interfering with the function of tumor-associated dendritic cells [53]. The XBP1s and ATF6α target, P58IPK, has been linked to survival of malignant cells facing ER stress, mediating an adaptive response to chronic UPR signaling [54].

PERK signaling has been reported to both inhibit and promote tumor growth. PERK deficient breast cancer cells display impaired cell growth and increased ROS production [55]. In contrast, inactivation of PERK in a murine model of medulloblastoma promotes tumor growth [56]. Furthermore, the oncoprotein MYC was shown to induce PERK and thereby promote cell survival through cytoprotective autophagy [57]. A recent study also connects ER homeostasis to epithelial-to-mesenchymal transition (EMT), showing that cells undergoing EMT have elevated BiP expression and activated PERK-eIF2α pathway that may be responsible for the increased secretory potential and sensitivity to ER stress inducers [58]. Other studies, however, have shown that CHOP induces apoptosis in cancer cells [54]. A PERK-ATF4 dependent miRNA, miR211, downregulates CHOP expression and promotes tumor cell survival in breast cancer [59]. These examples highlight the diverse effects of PERK signaling on apoptosis.

The outcome of UPR signaling in different cancer types is variable and depends on a multitude of factors such as tissue of origin, tumor stage and the tumor microenvironment. In addition, the heterogeneity of tumor cells represents a challenge when studying the consequence of UPR signaling in cancer.

Genetic manipulation of UPR components *in vivo* has revealed a spectrum of phenotypes, corroborating the fact that these signaling pathways are essential for the normal functioning of a number of organs. Similarly, naturally occurring mutations in the UPR sensors in humans lead to a range of malignancies such as metabolic diseases and cancer [60]. For example, somatic mutations in *IRE1* have been detected in several human cancers, such as hepatocellular carcinoma, glioblastoma, ovarian cancer, lung cancer, renal cancer and gastric cancer [61-63]. Furthermore, *XBP* mutations have been found in samples from patients with multiple myeloma [64, 65]. Data from the Catalogue of Somatic Mutations in Cancer (COSMIC) database have revealed a range of different mutations in *IRE1, PERK* and *ATF6* which show specific mutation patterns and tissue distribution [12]. The biological effects of the different mutations on tumor progression are at present not clear; however, a number of the IRE1α mutations identified in human cancers appear to provide a survival advantage that can still splice XBP1, but cannot induce RIDD [13].

## UPR IN PROSTATE CANCER

Prostate cancer (PCa) represents a major health issue worldwide as the most commonly diagnosed cancer after skin cancer and the second leading cause of cancer deaths among men in the western world [66]. It is estimated that approximately one of seven men will be diagnosed with PCa in their lifetime. One of the most central signaling pathways in all stages of PCa is that mediated by androgens. If the disease is not organ confined and thus curable by surgery alone, the standard therapeutic option for PCa is androgen ablation leading to an initial regression of the tumor in the vast majority of the cases. However, in most cases the tumors relapse into castration resistant PCa (CRPC) for which there is currently no curative treatment available [67, 68].

Being a major secretory organ, the prostate is particularly reliant on proper functioning of the ER and is vulnerable to agents or conditions that cause ER stress. It has been suggested that ER stress and UPR activation are involved in PCa as earlier studies indicated a negative correlation of UPR marker gene expression and PCa progression in model systems *in vitro*. For example, XBP1 was found downregulated in PCa compared with normal prostate and displayed an inverse correlation with pathological grade [69]. This study, however, did not differentiate between the spliced and unspliced forms of XBP1. The three UPR branches were also found to be downregulated during prostate tumorigenesis in both the *NKX3.1:PTEN* mutant and MYC-overexpression PCa mouse models [70].

In contrast, a number of studies have pointed to a positive association between ER/UPR markers and PCa development, mainly driven by androgens and androgen receptor (AR) signaling. Androgens regulated the expression of different ER stress associated genes in PCa, including N-myc downstream-regulated gene 1 protein (*NDRG1)*, protein disulfide isomerase-related protein (*PDIR/PDIA5*), homocysteine-responsive ER resident ubiquitin-like domain member 1 protein (*HERPUD*), and oxygen-regulated protein 150 (*ORP150)* [71]. Furthermore, gene expression profiling of dihydrotestosterone (DHT)-treated ventral prostates from rats revealed that genes whose products are involved in protein synthesis, degradation and processing are differentially expressed. These included calnexin, calreticulin, BiP, GRP94 and PDI [72]. Another study found that expression of genes associated with protein synthesis, folding and secretion, such as ribosomal proteins and seminal vesicle secretion proteins, were significantly induced in mouse prostates treated with DHT further strengthening the connection between androgen signaling and ER function [73].

In spite of the pivotal role of AR signaling throughout all stages of PCa development, its role in regulation of canonical UPR arms was not understood until recently. We found that androgens activated the IRE1α-XBP1 arm and simultaneously inhibited the PERK-eIF2α pathway [74]. Moreover, activated AR directly bound in the vicinity of *IRE1*, as well as XBP1s targets *RAMP4* and *EDEM1* genes, in PCa cells. Coupled with the complete loss of androgen regulation upon AR knockdown, these data document that these genes are direct AR targets. Consistent with these findings, AR and UPR gene expression were correlated in human PCa samples whereas XBP1s protein expression is significantly increased in cancer compared to normal prostate [74].

In contrast to the findings on the IRE1α arm, the mechanisms behind androgen-mediated inhibition of PERK-eIF2α signaling is not clear at present. Whereas PERK activation and thus eIF2α phosphorylation is downregulated by androgens, expression of downstream targets ATF4 and CHOP were increased at the protein level [74]. This is despite the fact that *ATF4* mRNA expression is not significantly affected whereas *CHOP* mRNA expression is decreased by androgens. One possible explanation of these observations is that upon dephosphorylation of PERK and eIF2α by androgen treatment, in PCa cells there is a general increase in protein synthesis which compensates for the effects observed at the mRNA level. Alternatively, CHOP may act as a survival factor in PCa as was suggested in some other settings [32]. In addition, in different PCa cell lines regulation of the PERK pathway may be different. For example, in the CRPC model 22Rv1 cells androgens activate eIF2α phosphorylation [75]. Further analyses are thus required to uncover the details in the regulation of the PERK pathway by androgens in PCa cells and the corresponding phenotypic outcomes.

## POTENTIAL FUNCTIONS OF UPR IN PROSTATE CANCER

Compared to PERK and ATF6, there is relatively more data on the functional role of IRE1α-XBP1s signaling in PCa. In LNCaP cells, depletion of IRE1α or its downstream target XBP1 leads to inhibition of cell growth *in vitro* and *in vivo*, a likely consequence of decreased proliferation and increased apoptosis [74]. Targeting this arm using toyocamycin, a small molecule drug inhibiting IRE1α kinase activity and *XBP1* splicing, profoundly inhibits both LNCaP and VCaP cell growth *in vitro* as well as tumor formation *in vivo* [74]. Increased *XBP1* splicing in the AR negative PC3 cells by treatment of Sphingosine 1-phosphate, a bioactive lysophospholipid, induces autophagy and exerts a cytoprotective effect [76]. Consistently, inhibition of IRE1α kinase activity and subsequent *XBP1* splicing impairs proliferation of these cell lines *in vitro* [77]. However, the exact role of the IRE1α-XBP1s pathway in AR-negative cell lines is not clear at present and further investigation is warranted. In summary, the IRE1α-XBP1s arm plays a pro-survival role in PCa suggesting that targeting IRE1α signaling may be a novel therapeutic strategy for PCa.

In contrast to the IRE1α arm, little is known about the functions of PERK-eIF2α and ATF6 in PCa progression. ATF4 expression has been shown to be induced by leucine or androgen deprivation in PCa cells [78]. ATF4 subsequently activates transcription of L-type amino acid transporter 1 (*LAT1*) which is important for leucine uptake and cell growth [78]. Furthermore, ATF4 mediates the pro-survival role of six-transmembrane protein of prostate 2 (STAMP2), a protein which impacts both cell growth and cell death pathways in PCa cells [79]. In addition, ATF4 expression is significantly upregulated in CWR22R refractory tumors, implicating it in progression to CRPC [79].

In contrast, there is currently no direct evidence that links the ATF4 target CHOP to survival or apoptosis in PCa cells. CHOP directly induced the expression of death receptor 5 (DR5) in response to either tunicamycin or acetyl-keto-β-boswellic acid treatment, both of which trigger apoptosis in PCa cells [80, 81]. Further work is required to delineate the role of PERK signaling in PCa.

## ER CHAPERONES IN PROSTATE CANCER

Expression of molecular chaperones with cytoprotective roles is elevated in several types of cancer [82] including in PCa (for a review, see [83]). The most studied ER chaperone in PCa cells is BiP. Increased BiP expression is correlated with greater risk of PCa recurrence and worse survival [84]. Consistently, double knockout of *Bip* and *Pten* in prostates of mice completely reverts the invasive adenocarcinoma phenotype normally observed upon *Pten* deletion, further establishing BiP as a key regulator of PCa progression [85]. Notably, the intracellular localization of BiP seems to be critical for its function; BiP has been detected on the cell surface in several cancers, including PCa, but not on normal cells, suggesting that it is a cancer-specific cell surface marker [86]. Translocation of BiP from the ER lumen to the cell surface is implicated in the hormonal resistance of PCa as well as in breast cancer cells, which may be mediated by enhanced PI3K signaling [87].

A variety of strategies have been employed to specifically target cell surface BiP, such as siRNA, antibodies, peptides, fusion proteins and nanoparticles, all resulting in reduced growth and increased apoptosis both *in vitro* and *in vivo* [88-92]. A recent study confirmed that targeting surface BiP with the peptide ligand SNTRVAP suppressed castration-resistant osteoblastic bone metastases *in vivo* [93]. In addition, prostate apoptosis response 4 (PAR-4), a pro-apoptotic protein secreted by cancer cells, was shown to bind to cell surface BiP and induce apoptosis of PCa cells [94]. Another study has linked androgen-induced impairment of autophagy to an increase in BiP protein levels independent of PERK-eIF2α-ATF4 pathway activation; however, the mechanism behind this effect is currently unclear [95]. These studies suggest that BiP may be a biomarker and therapeutic target for PCa, but further investigations are required to evaluate this possibility.

In addition to BiP, expression of several other molecular chaperones, such as heat shock protein 27 (HSP27) and HSP90, have been associated with aggressive human PCa [96-98]. Overexpression of HSP27 alleviates MG132-induced UPR and inhibits apoptosis of PC3 cells [99]. HSP27 also drives EMT and metastasis in PCa [100]. Furthermore, combining the non-invasive low energy focused ultrasound (LOFU) with a non-toxic dose of the HSP90 inhibitor 17AAG has displayed promising results in PCa xenografts by shifting the pro-survival UPR to a pro-apoptotic response [101]. Chemical inhibition of both HSP90 and HSP27 exerts a synergistic inhibitory effect on LNCaP cell growth *in vitro* and *in vivo* [102]. In summary, ER chaperones appear to be key players in tumor survival and treatment resistance, making them potentially valuable therapeutic targets.

## OTHER ER-ASSOCIATED PROTEINS IN PROSTATE CANCER

An important biosynthetic function of the ER is glycoprotein production. The glycosylation process involves the addition of an oligosaccharide consisting of N-acetylglucosamine, glucose and mannose moieties to the NH2-side chain of asparagine and is termed N-linked glycosylation. Several proteins involved in ER N-glycosylation have been linked to PCa. For example, the ER-localized protein ectonucleoside triphosphate diphosphohydrolase 5 (ENTPD5), a downstream target of AKT, is important for N-linked glycosylation and increases aerobic glycolysis. ENTPD5 is elevated in PCa and confers a survival advantage to PCa cells, connecting ER function to PCa metabolism [103]. Another example is UDP-N-acetylglucosamine pyrophosphorylase 1 (UAP1), an enzyme of the hexosamine biosynthetic pathway involved in N-glycosylation, that is androgen regulated and highly overexpressed in PCa. Increased levels of UAP1 in PCa cells provides a growth advantage as it confers resistance of PCa cells towards inhibitors of N-linked glycosylation, such as tunicamycin and 2-deoxyglucose [104]. Furthermore, inhibition of N-linked glycosylation in PC3 and DU145 cells, either by tunicamycin or glycosydase, leads to the generation of a lower molecular-weight pattern of death receptor 4 associated with apoptosis [105]; however, how this isoform triggers apoptosis is not known. Recently, tumor suppressor candidate 3 (TUSC3), a member of the oligosaccharyltransferase complex, has also been implicated in N-glycosylation and growth of PCa cells [106]. Loss of TUSC3 leads to increased proliferation, viability, migration, and invasion of DU145 and PC3 cells, which is accompanied by elevated N-glycosylation and AKT phosphorylation, indicating that TUSC3 exerts its tumor suppressor activity by influencing these processes [106]. The involvement of these proteins in the ER glycosylation processes in PCa cells is particularly intriguing given the altered glycosylation patterns found in PCa [107].

Another relevant ER stress associated protein is N-myc downstream regulated gene-1 (NDRG1) which is a potent metastasis suppressor that plays a key role in regulating signaling pathways involved in PCa [108]. NDRG1 interactome map in LNCaP cells in response to androgens identified a group of ER chaperones and proteins involved in ER stress. Interestingly, BiP levels were unaffected upon NDRG1 knockdown, whereas GRP94 expression was decreased [109]. Speckle-type POZ protein (SPOP), a component of the CUL3-RBX1 E3 ubiquitin ligase complex, has been shown to interact with CHOP and induce its degradation. PCa cells expressing SPOP mutants showed a defect in inducing CHOP ubiquitination and underwent CHOP-mediated apoptosis [110]. These findings highlight the importance of ER-associated proteins in PCa, even if they may not be directly involved in ER stress. However, the majority of the findings to date on these proteins are mainly correlative and their possible functional impact in PCa is currently not known. Another ER-associated protein is PACE-4, a proprotein convertase that is shown to be upregulated in PCa tissues. A recent *in vitro* study using multiple PCa cell lines showed that siRNA knockdown of PACE4, led to apoptosis accompanied with increased PERK and eIF2α phosphorylation [111]. However, how PACE4 regulates these UPR arms and the molecular mechanisms of apoptosis in this context is currently not known.

Skp2, a critical component of the Skp2-SCF complex E3 ligase, is found highly expressed in numerous cancers including PCa [112]. Induction of eIF2α phosphorylation and ATF4 expression were credited to be partially responsible for the cellular senescence triggered by loss of Skp2 in mouse embryonic fibroblasts [113]. Interestingly, BiP and phospho-PERK level remained unchanged upon Skp2 loss, suggesting that other eIF2α kinases and compensatory mechanisms may be involved. Another study using a panel of human PCa cell lines revealed an inverse relationship between Skp2 and ATF4 expression upon caffeic acid phenethyl ester treatment, the chief extract from honeybee hive propolis [114]. However, whether Skp2 plays a role in the context of ER stress and UPR in PCa is largely unknown.

An active field of investigation in PCa in recent years has been the interplay between ER stress and autophagy. Autophagy is another typical adaptive mechanism by which the cells react to metabolic, toxic, and even infectious stressors [115]. It is dynamically regulated by a number of factors, for instance the PI3K-AKT-mTOR signaling pathway which is frequently found activated in human PCa due to the loss of PTEN [68, 115-117]. Various agents and chemicals that induce ER stress have also been shown to induce autophagy in PCa cells [118, 119]. The ER stress-triggered autophagy appears to play a protective role under these conditions by clearing polyubiquitinated protein aggregates and reducing cellular vacuolization [118]. On the other hand, acutely-stimulated ER stress has been shown to induce expression of the proapoptotic protein, PAR-4, which switches protective autophagy to apoptosis in androgen-independent PCa cells by inhibiting autophagy-related proteins BCL2 and BECLIN-1 [120]. In turn, disruption of autophagy may give rise to ER stress through feedback mechanisms. For instance, in a prostate-specific *Pten*-deficient mouse model, additional knockout of autophagy-related-7 (*Atg7*) markedly delayed tumor development under both castration-naïve and castration-resistant conditions [121]. The double knockout phenotype displayed impaired autophagy and increased ER stress, underscoring the importance of protein homeostasis in PCa progression [121]. There are a number of detailed recent reviews on the possible roles of PI3K-mTOR-Akt pathway as well as autophagy in PCa [115, 117, 122].

## TARGETING THE ER STRESS RESPONSE FOR PROSTATE CANCER THERAPY

Given the significance of maintaining proteostasis in cancer cells, targeting the ER homeostasis is emerging as a new therapeutic strategy [123], also in PCa. In addition to the compounds exemplified above targeting the IRE1α arm, various small molecule drugs and chemical extracts that disrupt ER homeostasis *in vitro* in PCa cells have been identified. For example, selenium and its metabolites had anti-cancer activity on PCa cells through, at least in part, activation of ER stress and subsequent induction of apoptosis, an effect which was attenuated by BiP overexpression [124]. In line with this, BiP knockdown greatly enhanced the growth inhibition of PC3 cells by selenium [125]. Metformin, a first-line anti-diabetic drug, has been shown to decrease the risk of PCa in people who use it for metabolic disturbances [126]. Research has shown that this may be mediated by the activation of the miR-708-5p/neuronatin pathway, which subsequently leads to ER stress-induced apoptosis [127]. However, combined application of ER stress inducers should be carefully examined, as the outcome may not always be detrimental. This is exemplified by a recent study in PC3 cells, where epigallocatechin gallate (EGCG), a major bioactive green tea polyphenol known to induce ER stress, failed to further promote cell death triggered by either bortezomib or MG132. Instead, EGCG activated cytoprotective autophagy, reduced CHOP levels and thereby protected from bortezomib-caused ER stress and apoptosis [128].

A number of natural compounds that have anti-cancer activities interfere with ER function and induce PCa cell death. In addition, several enzyme inhibitors have been shown to trigger ER stress and induce apoptosis in PCa. Molecules that have been identified to date that target ER homeostasis are summarized in Table 1. However, it should be noted that the data are mainly from either *in vitro* studies or limited preclinical models; thus, additional translational studies investigating these compounds in an *in vivo* setting are needed.

**Table 1:** Molecules targeting ER homeostasis in prostate cancer

Molecule	Origin	Phase	Readout	Refs
Toyocamycin	Actinomycete	Preclinical, LNCaP and VCaP	Inhibition of *XBP1* splicing	[74]
N-butylidenephthalide	*Angelica sinensis*	Preclinical, LNCaP	Induction of CHOP, IRE1 and BiP	[129]
Tanshinone IIA	*Salviae Miltiorrhizae Radix*	Preclinical, LNCaP	Induction of CHOP, IRE1 and BiP	[130]
SMI-4a	Pim kinase inhibitor	Preclinical, LNCaP	Activation of eIF2α, ATF4, CHOP and induction of *XBP1* splicing	[131]
Curcumin	Turmeric	Preclinical, PC-3M	Induction of IRE1, eIF2α, CHOP and BiP	[132]
Clofoctol	Antibiotic for upper respiratory tract infections	Preclinical, PC3	Activation of IRE1, PERK and ATF6 pathways	[133]
Monascuspiloin	Monascus pilosus M93-fermented rice	Preclinical, PC3	Activation of IRE1 and eIF2a	[134]
Marchantin M	Bryophytes	Preclinical, PC3	Induction of CHOP and BiP	[135, 136]
Nelfinavir	HIV protease inhibitor	*In vitro*, PC3 and DU145	Activation of ATF6, BiP and S2P target gene expression	[137]
Shikonin	*Lithospermum erythrorhizon*	*In vitro*, PC3 and DU145	Activation of PERK, eIF2α, CHOP and BiP	[138]
Methylseleninic acid	Selenium	*In vitro*, PC3	Activation of PERK, eIF2α, CHOP and BiP	[124]
Celastrol	Autoimmune diseases, chronic inflammation, asthma and neurodegenerative disease	*In vitro*, PC3	Activation of IRE1, PERK and BiP	[139]
Polyphenon E	Green tea	*In vitro*, PC3	Activation of CHOP	[140]
Diindolylmethane	Indole-3-carbinol	*In vitro*, DU145	Activation of IRE1	[141]
Capsaicin	Hot chilli peppers	*In vitro*, PC3	Activation of eIF2α, ATF4 and CHOP	[142]
4,5,6,7-tetrabromobenzotriazole	Protein kinase CK2 inhibitor	*In vitro*, PC3	Activation of CHOP	[143]
VN/124-1	17A-hydroxylase/17,20 lyase inhibitor	*In vitro*, PC3	Activation of eIF2α and BiP	[144]
Proteasome inhibitor-I		*In vitro*, PC3	Inhibition of IRE1 phosphorylation and induction of CHOP	[145]
Triptolide	*Tripterygium wilfordii* Hook F.	*In vitro*, PC3	Inhibition of BiP and activation of IRE1α, PERK and eIF2α	[146]
Metformin	First-line antidiabetes drug	*In vitro*, C4-2B	Induction of miR-708-5p and inhibition of neuronatin	[127]

## CONCLUSIONS

The data that have accumulated in the last few years indicate that ER stress and the UPR play an important role in PCa. Among these intriguing findings are the androgen regulation of UPR activation, the functional roles of different UPR components, as well as the differential regulation of ER chaperone expression. These data suggest that targeting the adaptive survival aspects of the UPR and interfering with ER homeostasis is potentially a novel and promising approach for PCa therapy.
